# Biofilms facilitate cheating and social exploitation of β-lactam resistance in *Escherichia coli*

**DOI:** 10.1038/s41522-019-0109-2

**Published:** 2019-11-29

**Authors:** Elli Amanatidou, Andrew C. Matthews, Ute Kuhlicke, Thomas R. Neu, James P. McEvoy, Ben Raymond

**Affiliations:** 1 Department of Biological Science, Royal Holloway University of London, Egham, Surrey UK; 2 Centre for Ecology and Conservation, University of Exeter, Penryn campus, Penryn, Cornwall UK; 3 Helmholtz Centre for Environmental Research, Magdeburg, Germany; 4 Present Address: Food Standards Agency, London, UK

**Keywords:** Biofilms, Pathogens, Evolution, Antimicrobials

## Abstract

Gram-negative bacteria such as *Escherichia coli* commonly resist β-lactam antibiotics using plasmid-encoded β-lactamase enzymes. Bacterial strains that express β-lactamases have been found to detoxify liquid cultures and thus to protect genetically susceptible strains, constituting a clear laboratory example of social protection. These results are not necessarily general; on solid media, for instance, the rapid bactericidal action of β-lactams largely prevents social protection. Here, we tested the hypothesis that the greater tolerance of biofilm bacteria for β-lactams would facilitate social interactions. We used a recently isolated *E. coli* strain, capable of strong biofilm formation, to compare how cooperation and exploitation in colony biofilms and broth culture drives the dynamics of a non-conjugative plasmid encoding a clinically important β-lactamase. Susceptible cells in biofilms were tolerant of ampicillin—high doses and several days of exposure were required to kill them. In support of our hypothesis, we found robust social protection of susceptible *E. coli* in biofilms, despite fine-scale physical separation of resistant and susceptible cells and lower rates of production of extracellular β-lactamase. In contrast, social interactions in broth were restricted to a relatively narrow range of ampicillin doses. Our results show that β-lactam selection pressure on Gram-negative biofilms leads to cooperative resistance characterized by a low equilibrium frequency of resistance plasmids, sufficient to protect all cells.

## Introduction

It is now widely accepted that many microbes lead social lives and share social traits, in particular secreted metabolites that act as ‘public goods’, which are costly for individuals to produce but which provide group level benefits.^1–5^ Public goods in microbes include siderophores,^6^ toxins and their antidotes,^5,7^ as well as quorum-sensing molecules and quorum-regulated virulence factors.^2,8^ Enzymes that confer antibiotic resistance, such as the β-lactamases that are responsible for detoxifying penicillins, can also be considered as public goods.^9–12^ β**-**lactamase secretion is not essential for cooperation, since even non-secreted detoxifying enzymes can be cooperative traits by providing an antibiotic-free space as a public good.^13^ In this context, antibiotic-susceptible bacteria may be socially exploitative and possess genotypes that allow them to freeload on the individually costly effort of others.

Antibiotic resistance is a major public health concern, and β**-**lactam resistance has particular clinical importance.^14,15^ Understanding how antibiotics may or may not select for resistant genotypes is therefore valuable, especially since social exploitation of β-lactamases could moderate overall selection for resistance. Here, a major issue is whether experiments on social dynamics in the laboratory are of relevance to other ecological or physiological contexts. An important step would be to understand the implications of diverse levels of cooperation in different experimental systems. Notably, non-producer/cooperator dynamics can differ enormously between experiments conducted in liquid and solid media. Many of the earlier experiments on social interactions in bacteria used nutrient-rich broth culture, which can increase the availability of public goods and facilitate social exploitation.^6,9^ In liquid media, β-lactamase-producing *Escherichia coli* can protect susceptible strains efficiently, implying that the production of the enzyme is a public good in bacterial communities.^9–11^ The proportion of resistant cells in broth can equilibrate to values as low as 25% with ampicillin at 100 µg/ml, a dose 25× higher than the minimum inhibitory concentration (MIC).^11^ This equilibrium proportion was found to depend on the ratio of the antibiotic concentration to the initial total cell density.^11^ In contrast, detoxification by resistant cells is relatively slow on solid media, and because β-lactams are bactericidal, only metabolically inactive persister cells, a tiny proportion of genetically sensitive cells, are able to exploit β-lactamases produced by resistant bacteria.^12^

A better understanding of bacterial sociality in diverse contexts is needed to understand competition between bacteria resistant and susceptible to β-lactams. Bacterial growth in the environment or in hosts rarely resembles growth either on an agar plate or in broth. Growth in biofilms, however, is commonplace and is a widely cited paradigm for in vivo growth conditions.^16,17^ This makes biofilms an important testing ground for hypotheses involving bacterial sociality, particularly because distinct bodies of work lead to opposing predictions. Biofilms could impede cooperation if the additional physical structure in biofilms limits distribution of public goods and restricts social exploitation.^18,19^ Moreover, relatedness is increased in spatially structured environments, so that opportunities for non-producers to exploit the resource of others might be reduced in biofilms.^18–20^

In contrast, physiological considerations suggest that biofilms might facilitate cooperation in terms of resistance based on detoxifying β-lactamases. Given that biofilms are highly tolerant of antibiotics, and that tolerance of the bactericidal effects of β-lactams in susceptible cells can facilitate exploitation of resistant cells,^12^ we would predict that susceptible cells might be better able to survive and benefit from the action of resistant bacteria in biofilms, thereby moderating selection pressure for resistance. Moreover, widespread physiological tolerance of antibiotics might extend the benefits of β-lactamases to a wider proportion of the population than fully dormant persisters, which typically make up only a very small proportion of the bacterial population.^21^

In line with previous studies, we examined the implications of social interactions for the dynamics of a moderately costly non-conjugative plasmid encoding a β-lactamase.^9–12^ This set-up reflects reality—most β**-**lactamases in Gram-negative bacteria are plasmid-encoded,^15,22^ and the consumption of β**-**lactam antibiotics is known to increase the frequency of β**-**lactamase genes in *E. coli* and of clones characterized by plasmid-encoded resistance.^23,24^ In addition, β**-**lactamases themselves impose very small fitness costs so that the fitness burden associated with the acquisition of resistance primarily derives from the plasmid backbone.^25^ Although the equilibrium level of social β**-**lactamase resistance is predicted to depend only weakly on fitness costs,^11^ the fitness cost of the plasmid drove our system to equilibrium in a practical timeframe. In any case, since the dynamics of resistance in *E. coli* will primarily depend on the prevalence of plasmids outside of the laboratory, a plasmid-based expression system was developed to explore how selection, social cooperation and conflict determine the prevalence of resistance under controlled conditions.

The aim of this study was to investigate the extent of cooperative detoxification of β-lactams in biofilm and liquid cultures, and specifically to test the hypothesis that increased tolerance of antibiotics in mature biofilms would facilitate social protection of susceptible cells. We predicted that biofilm tolerance would contribute to the dynamics of strain competition and the overall antibiotic resistance of the population. Experiments were conducted using a recently isolated wild-type (WT) *E. coli* strain that is a strong biofilm former. Using a clinically relevant β-lactamase, *bla* _*CTX-M-14*_^26,27^ we examined the extent of cooperation by measuring the competitive dynamics and fitness of susceptible bacteria at different proportions of resistant cells over a range of antibiotic doses. In serial transfer experiments, we identified the equilibrium proportion of resistant bacteria that protected biofilms and liquid media from a β-lactam antibiotic. We investigated the ecological conditions for exploitation and cooperation by assessing the magnitude of intracellular and extracellular β-lactamase activity in broth and biofilms, and the extent of genotypic mixing at a fine spatial scale with confocal laser scanning microscopy (CLSM). Our results indicate that the survival of cells in established biofilm containing both resistant and susceptible strains may depend on social interactions in combination with physiological tolerance.

## Results

### Characterization and selection of WT biofilm forming *E. coli*

**Fig. 1:**
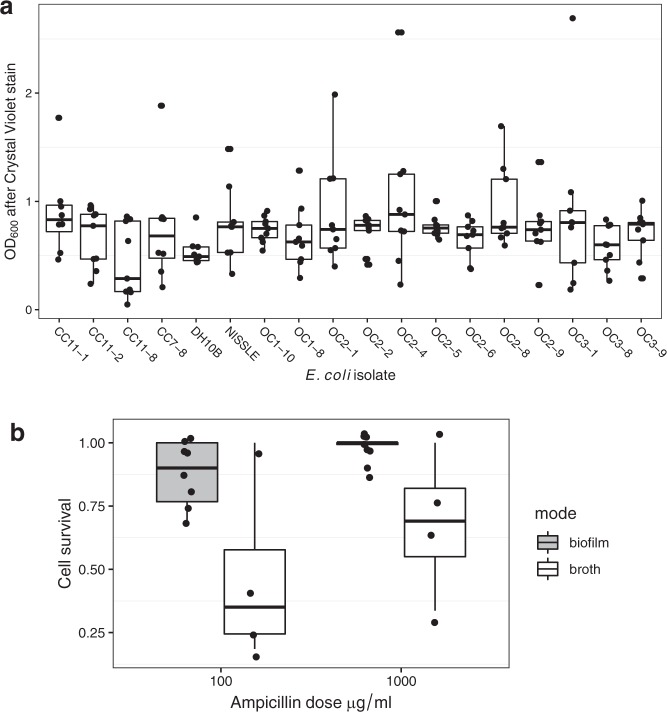
Biofilm formation, structure and short-term antibiotic tolerance. **a** Biofilm growth of *E. coli* strains measured by a micro-titre plate crystal violet assay. Assays compared recent field isolates with a standard laboratory strain (DH10β) and the probiotic Nissle as a positive control. Data are from three experiments, each using with three replicates. Isolate cc11-1 was used in all future experiments and had a significantly increased biofilm production relative to DH10β (*t* *=* 2.14, *P* = 0.03). **b** Boxplots showing survival of cells recovered from broth and biofilm culture after a 2 h exposure to ampicillin. The difference between the growth modes was significant at *P* < 0.01. All boxplots show medians, first and third quartiles and 1.5 × the interquartile range as whiskers, and are overlaid with jittered raw data.

Screening recently collected *E. coli* isolates identified a range of strains that could produce biofilms as effectively as *E. coli* Nissle, and thicker than our weakly biofilm-forming laboratory strain DH10β (Fig. 1a, effect of strain-Likelihood ratio = 34.2, df *=* 17, *P* = 0.0079). Isolate cc11-1 was selected for further experimental work because it was transformable, non-haemolytic and carried no native β-lactam resistance. This isolate produced a strong, firmly attached biofilm when grown on filter membranes.

We recovered cells from 24 h biofilm and broth cultures and challenged them with high doses of ampicillin for 2 h in order to compare the tolerance of this antibiotic between these two growth modes. Cells grown in biofilms had much higher tolerance of antibiotics that broth-cultured *E. coli* (Fig. 1b, *F*_1,21_ = 11.7, *P* = 0.0028) although the dose of ampicillin in this experiment did not affect survival over this short period (Fig. 1b, *F*_1,20_ = 2.91, *P* = 0.104).

### Microscopy

**Fig. 2:**
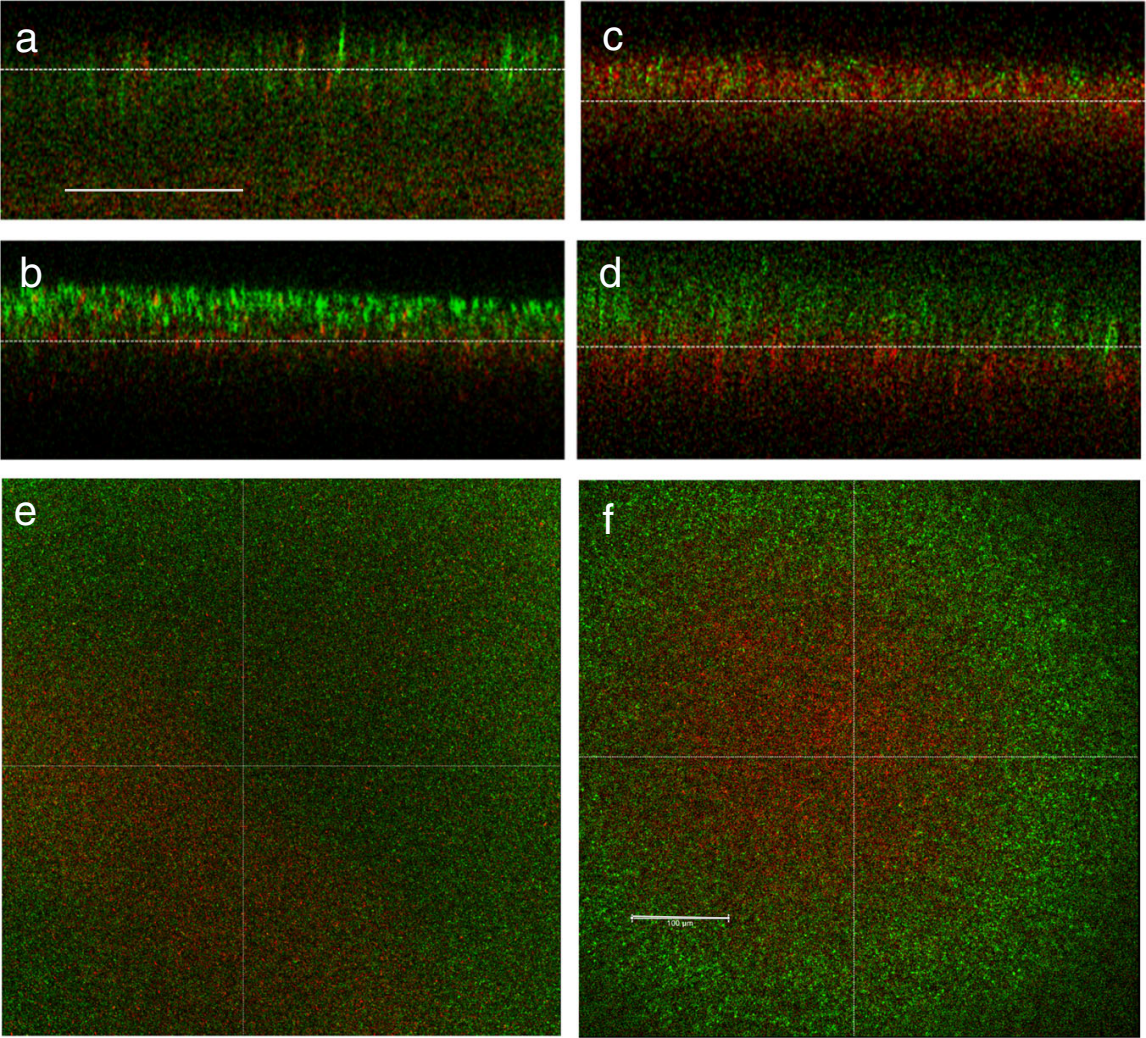
Multichannel confocal laser scanning microscopy of biofilm stucture. Experiments used cc11-1 with antibiotic-resistant plasmids (red cc-11-1 CrimCTXM) in competition with plasmid-free cells marked chromosomally with a green-fluorescent protein (cc11-1CyanΔLacZ). Experimental conditions duplicated those in the biofilm transfer experiment. Panels **a**–**d** show vertical cross-sections from z-stacks, the bottom of the image corresponds to the bottom of the biofilm, i.e. the edge in contact with the filter membrane. Panels **e**, **f** show the top view of biofilms. Panels **a**, **b**, **e** used 50:50 mixtures of plasmid carriers and plasmid-free cells at set-up, **a** shows the first biofilm inoculated from broth, **b**, **e** show biofilms grown after one transfer. Panels **c**, **d**, **f** used 90:10 mixtures of plasmid carriers and plasmid-free cells at set-up, **c** shows the first biofilm inoculated from broth, **d, f** show biofilms grown after one transfer. Scale bars are 100 μm and are common for the vertical cross sections **a**–**d** and top views **e**, **f**.

We characterized biofilm structure of plasmid-carrying and plasmid-free cc11-1 grown in our static filter-membrane experimental system. We tested the assumption that growth in a biofilm will produce spatially localized growth of plasmid carriers and non-carriers. CLSM showed that these genotypes had fine-scale spatial structure (Fig. 2a–f). However, imaging across vertical cross sections revealed structure across the *z*-axis consistent with cells dividing and growing away from the base of the biofilm, which is in contact with the membrane. Populations on the top surface of the biofilms were increasingly dominated by the faster growing plasmid-free cells, especially after one round of cell transfer from a disrupted biofilm to a new biofilm (Fig. 2b, d). After one round of transfer, the top views covering nearly the entire biofilms also show increased dominance of plasmid-free cells at the biofilm edges (Fig. 2e, f).

When experiments were repeated with resistant and susceptible cells with similar growth rates (that were both plasmid carriers), evidence for broad scale structuring across the vertical axis was weaker (Supplementary Materials). Finer scale examination showed resistant and susceptible cells commonly formed clusters separated by voids and the absence of cells in the voids was confirmed by rhodamine staining (Supplementary Fig. 1).

### Cell abundance in transfer experiments

**Fig. 3:**
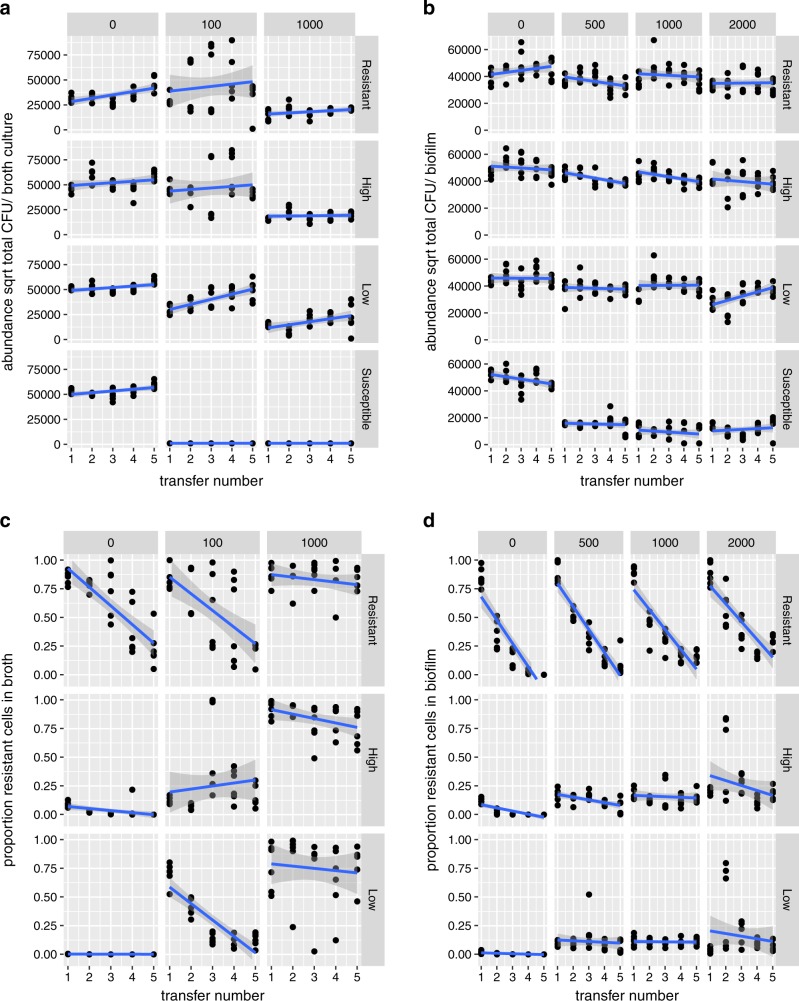
Population dynamics of β-lactam resistant (cc-11-1 CrimCTXM) and susceptible *E. coli* cc-11-1 in transfer experiments in broth and biofilms. Results obtained at different ampicillin concentrations (top strip labels, μg/ml) are shown in each column. Data obtained from different starting frequencies of resistant bacteria are shown in each row: Resistant, high, low and susceptible, indicating initial frequencies of 100%, 50%, 2% and 0% cc-11-1 CrimCTXM. Data points represent independent replicates. Bacterial abundance is expressed per culture, as biofilm volumes were not controlled. **a** Total bacterial abundance in broth cultures; **b** total bacterial abundance in biofilms; **c** proportion of resistant bacteria in broth cultures; **d** proportion of resistant bacteria in biofilms.

Here, we examined the dynamics of β-lactam resistant and susceptible cells in order to test the hypothesis that biofilms would facilitate more social exploitation than liquid culture conditions. In LB, ampicillin doses of 100 and 1000 μg/ml were lethal to cultures containing only susceptible cells (Fig. 3a), while doses of 500, 1000 and 2000 μg/ml were merely inhibitory to susceptible-only biofilms (Fig. 3b).

In liquid cultures, ampicillin had the consistent effect of reducing bacterial abundance for all resistance frequency treatments (dose ∗ frequency interaction df = 3, LR = 2.35, *P* = 0.51; dose main effect df = 3, LR = 62.3, *P* < 0.0001; Fig. 3a). Initial resistance frequency predominantly affected the overall abundance (frequency main effect df = 3, LR = 30.1, *P* < 0.0001; Fig. 3a), while abundance tended to gradually increase with transfer number in the whole experiment (df = 3, LR = 18.3, *P* < 0.0001), an effect consistent with an impact of plasmid carriage on overall reproduction. Variability within time-points exposed to 100 μg/ml ampicillin was caused by fluctuating dynamics in abundance for particular replicates over time.

In biofilms, on the other hand, ampicillin only effectively suppressed the pure susceptible cultures (dose*frequency interaction df = 3, LR = 28.9, *P* < 0.0001). Overall, bacterial abundance was relatively stable between biofilm transfers. In the resistant biofilms without ampicillin and in the high dose/low resistance (LR) treatment abundance increased over time (three-way interaction, df = 3, LR = 7.98, *P* = 0.047). In contrast, bacterial abundance decreased over time in the high resistance (HR) treatment (slope estimate −1893 ± 494, *t* = −3.83, *P* = 0.001), here matched by a slight decline in the frequency of resistant cells between transfers.

### Proportion of resistant cells during the transfer experiments

When we initiated cultures with either a high or low frequency of resistant cells, the proportion of resistant bacteria was reduced to zero or very near zero in the absence of antibiotic, confirming that carriage of the resistance plasmid imposed a substantial cost (Fig. 3c, d). Initial loss of the resistance plasmid by segregation meant that fitness costs could reduce frequencies of resistance in experiments initiated with 100% resistant cells (R treatments).

In liquid culture, the dynamics of resistance were very different at the different ampicillin doses (three-way dose*frequency*time interaction, df = 2, LR = 12.5, *P* = 0.0019). Selection maintained resistance plasmids at a high and stable level of carriage at 1000 μg/ml ampicillin, while resistance showed negative frequency dependence at 100 μg/ml.

In biofilm transfers, the frequency of resistance declined very rapidly when starting from high initial frequencies but was stable in the low-frequency treatment (time*frequency interaction df = 2, LR = 187, *P* < 0.001), indicating strong negative frequency dependence at all doses. Resistance declined slightly more slowly as ampicillin dose increased (dose*time interaction df *=* 1, LR = 9.42, *P* = 0.021), evidence of the moderate selective power of ampicillin on resistance in this growth mode. The dynamics of resistance in biofilms were relatively insensitive to dose: pooling doses of 500, 1000 and 2000 μg/ml improved the fit of statistical models (AIC four-factor dose model −270.7, AIC two factor dose −309.9). In contrast, the specific ampicillin dose was very important for dynamics in broth, since pooling the doses of 100 and 1000 μg/ml substantially reduced the model fit (AIC three-factor dose model 41.0, AIC two-factor dose 82.9).

**Fig. 4:**
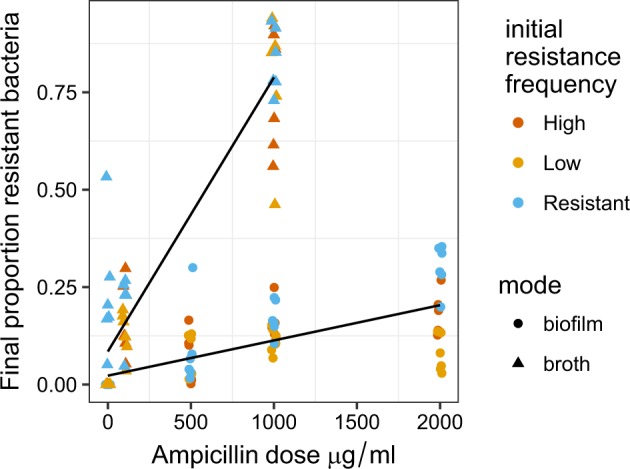
Final proportions of β-lactam-resistant *E. coli* cc11-1 after five transfers in liquid LB broth and biofilms. These are the endpoint data for the experiments described in Fig. 3, which varied dose of ampicillin and initial frequencies of resistant cells: resistant, high and low indicate initial frequencies of 100%, 50%, and 2% cc-11-1 CrimCTXM. Lines indicate fitted linear models and data points represent independent replicates. Antibiotic dose had a stronger impact on the final frequency of resistance in broth than in biofilm grown cells (growth mode*dose interaction *P* < 0.0001).

After five transfers, the growth mode (biofilm or broth) and ampicillin dose were the key determinants of the final frequency of resistance (Fig. 4; dose*mode interaction *F*_1,121_ = 36.3, *P* < 0.0001). Increasing dose strongly selected for increased resistance in liquid culture, while increasing dose only weakly, albeit significantly, selected for resistance in biofilms (slope = 0.12 ± 0.048, *t* = 7.59, *P* = <0.0001). When experiments were started with mixtures (LR or HR), the initial frequency had no significant impact on final frequency (*t* = −0.83, *P* = 0.41) indicating that these final frequencies approximated stable equilibria. Experiments initiated with 100% plasmid carrying cells (R treatment) retained a slightly higher proportion of resistant cells, implying that resistance had not quite reached a stable equilibrium in this treatment (estimate = 0.13 ± 0.14, *t* = 3.31, *P* = 0.0013).

### Relative fitness of susceptible cells in transfer experiments

**Fig. 5:**
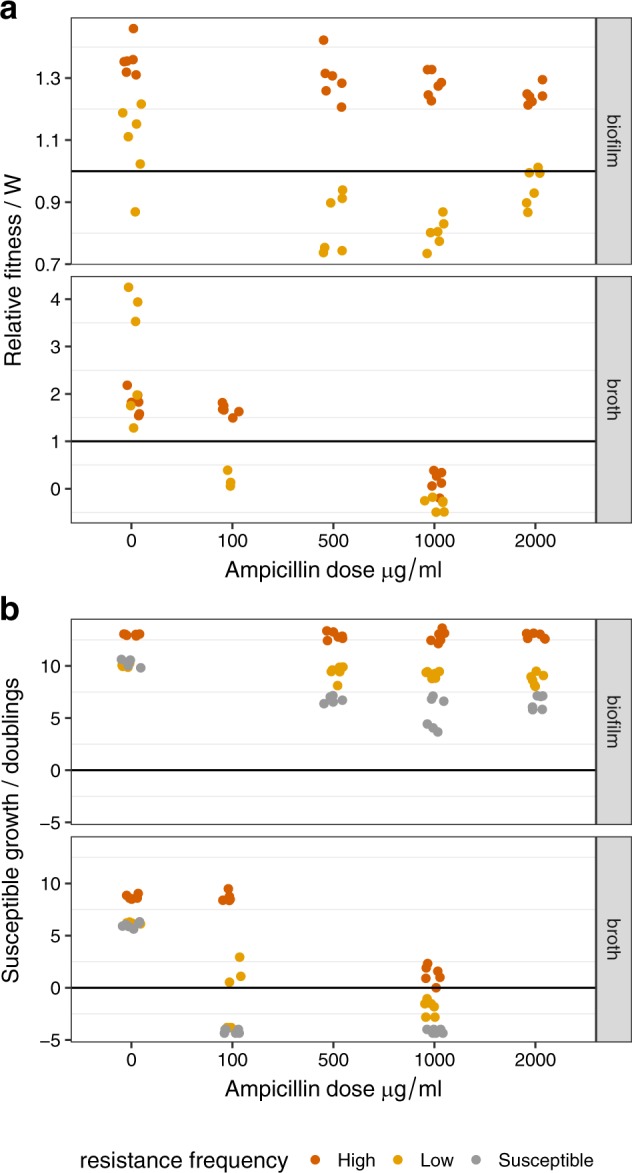
Fitness and reproductive rate assays for susceptible *E. coli* cc11-1 competing with β-lactam-resistant *E. coli* cc11-1 in LB broth and biofilms. **a** Relative fitness of susceptible cells at different doses of ampicillin. The line at *W* *=* 1 indicates equal fitness of resistant and susceptible bacteria. Dose has a much stronger effect on the relative fitness of susceptible cells in broth (dose ∗ mode interaction *P* *<* 0.0001). **b** The reproductive rate of susceptible cells, measured as number of doublings over the first experimental transfer. A negative reproductive rate indicates a decline in the abundance of cells, since inoculation and the line at 0 indicates no growth. In all panels the *x*-axis is categorical and data points represent independent replicates.

In the transfer experiment above, the relative fitness of the ampicillin-susceptible bacteria was calculated in mixtures over the first transfer. In support of our key hypothesis, susceptible cells were more effective exploiters of resistant bacteria in biofilms than in broth. In particular, they showed effective exploitation of β-lactamases (and higher fitness than resistant competitors) over a range of doses (Fig. 5a) whereas susceptible cells could only outcompete resistant cells in liquid culture at a dose of 100 μg/ml ampicillin. Thus, fitness in biofilms declined more slowly as the dose of antibiotic increased (mode*dose interaction, *F*_1,75_ = 95.8, *P* < 0.0001). Guided by theory, we expected the fitness of non-producers to be highest when public goods were more abundant, i.e. when the initial frequency of resistance bacteria was highest. Susceptible bacteria displayed this frequency-dependent fitness in both broth and biofilms (initial frequency*dose interaction *F*_1,76_ = 12.9, *P* = 0.0006) and could only outcompete resistant cells when resistance frequencies were high.

In order to quantify the effect of resistant cells on the growth of susceptible bacteria, we calculated the reproductive rate (bacterial doublings) of susceptible cells, as relative fitness is not meaningful for single strain cultures. As above we calculated growth in the first transfer, although subsequent transfers show qualitatively similar patterns. The reproductive rate of susceptible cells declined as dose increased, falling more markedly in broth than in biofilms (Fig. 5b; mode ∗ dose interaction *F*_1,118_ = 128, *P* < 0.0001). The presence and frequency of resistant cells also affected dose response (frequency ∗ dose interaction *F*_2,116_ = 6.64, *P* = 0.0019), predominantly because exposure to antibiotics reduced growth in biofilms composed only of susceptible cells relative to cultures containing resistant cells (post hoc contrast *t* = −5.66, *P* < 0.0001). Moreover, in biofilms containing a high frequency of resistant cells, the reproduction of susceptible cells was unaffected by antibiotic dose (test for non-zero slope *t* *=* 0.235, *P* = 0.81; Fig. 4b), indicating extremely effective detoxification in these growth conditions.

We also calculated the reproductive rate for resistant cells in order to test whether non-producing susceptible cells met the formal definition of cheaters, by lowering the fitness of the resistant producers.^28^ This analysis showed that the reproduction of resistant cells in biofilms was reduced in the presence of non-producers at all ampicillin doses *F*_2,68_ = 12.1, *P* < 0.0001), so that susceptible cells were formal cheaters in these biofilms. In contrast, there was no evidence that susceptible bacteria consistently reduced the reproductive rate of resistant bacteria in liquid culture (Supplementary Fig. 2).

### Dynamics and fitness under lethal dose conditions

While the transfer experiments described above investigated dynamics in biofilms exposed to strongly inhibitory ampicillin doses, here we tested whether similar dynamics would be seen in biofilms in which antibiotics were fully bactericidal. In these experiments, biofilms were exposed to 1000 μg/ml ampicillin for 3 days. The biofilms were moved daily to fresh plates, but were not disrupted as they were in the transfer experiments. This ampicillin concentration imposed lethal conditions on pure cultures of susceptible cells (Supplementary Fig. 3). As above, the relative fitness of susceptible cells was frequency dependent: susceptible cells could outcompete resistant bacteria provided there was a high frequency of detoxifying bacteria (*F*_1,22_ = 16.2, *P* = 0.0006) and this competitive interaction depended on the presence of antibiotic (dose*frequency interaction, *F*_1,21_ = 6.8, *P* = 0.016). Quantitatively, the relative fitness of susceptible cells at 1000 μg/ml ampicillin was very similar to that seen in the inhibitory dose experiments (Figs 4 and 5). However, the loss of resistant cells was slower at the higher dose: after 3 days in lethal conditions, final proportions of resistant cells were 0.10 ± 0.01 (SE), 0.20 ± 0.05 and 0.30 ± 0.03 in low, high frequency and resistant biofilms, respectively.

### β-lactamase activity in broth and biofilms

**Fig. 6:**
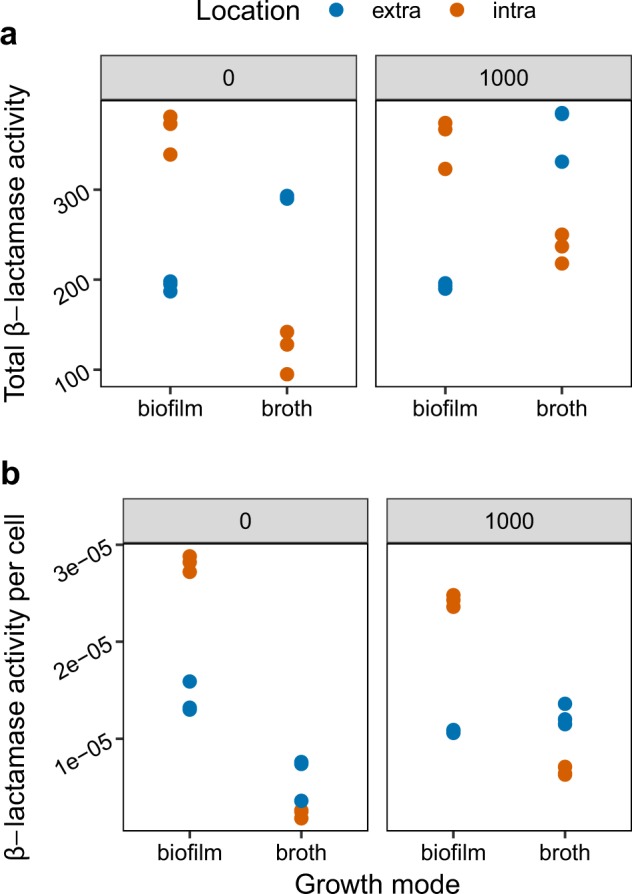
Intra-cellular and extra-cellular β-lactamase activity measured by nitrocefin assays of broth cultures and disrupted biofilms in the presence and absence of ampicillin. Top strip labels indicate ampicillin concentration in media in μg/ml, and activity is measured in absorbance units scaled to total culture volumes. **a** Broth cultures display more extra-cellular and less intra-cellular β-lactamase activity than cells from biofilms (*P* < 0.0001). **b** The balance between intra-cellular and extra-cellular β-lactamase activity per cell varies according to exposure to ampicillin (mode*exposure interaction *P* < 0.001. Data points represent independent replicates.

Here, we tested whether cheater dynamics and the increased fitness of susceptible cells in mixed biofilms might be explained by the increased availability of extra-cellular β-lactamase, the key public good in this study. However, biofilms demonstrated decreased total extra-cellular β-lactamase activity relative to broth (Fig. 6a; *F*_1,10_ = 22, *P* < 0.0001). In general, cells in biofilms retain more intra-cellular β-lactamase activity than those in broth (Fig. 6a, b). If we repeat these analyses after controlling for the total numbers of resistant cells in broth and biofilm, a slightly different picture emerges: we see a slight increase in extra-cellular β-lactamase activity *per cell* in biofilms than in broth (Fig. 6b; *F*_1,10_ = 13.1, *P* = 0.007). In the presence of antibiotics, however, this difference is reversed, and broth has the higher per cell β-lactamase activity (ampicillin*growth mode interaction *F*_1,8_ = 29.9, *P* = 0.0006).

## Discussion

Biofilms offer an ideal environment for microbial interactions because of their high cell density.^29^ Previous experiments suggest that the slower diffusion of public goods in biofilms might limit indirect fitness benefits, while spatial separation of clones will increase relatedness and decrease opportunities for social exploitation.^18,19^ However, we predicted that increased physiological tolerance of antibiotics would facilitate exploitation of β-lactamases in biofilms. Although we confirmed that biofilms produced spatially separate genotypes in this system, susceptible cells could more readily outcompete resistant cells in biofilms than in broth, as we predicted. We saw comparatively modest levels of segregation of genotypes at a fine spatial scale, consistent with a high level of inoculum.^30,31^ Nevertheless, the differences in growth rate of plasmid carrying and plasmid-free cells could produce substantial difference in relative abundance of these cells within biofilms and strong vertical structure, as seen in several other systems.^32^ In any case, modest levels of segregation are likely to have minimal impacts in this system, since β-lactams diffuse rapidly through biofilms.^33,34^ Importantly, susceptible cells could survive and retain high fitness over a wider range of antibiotic doses in biofilms than in liquid media. In biofilms, susceptible cells behaved as formal cheaters and reduced the reproductive rate of resistant bacteria when in competition.^28^ Moreover, no substantial difference in the fitness of susceptible cells was detected between strongly inhibitory and lethal antibiotic exposure. We saw low equilibrium frequencies of resistant cells in biofilms even at very high doses, indicating that relatively few resistant cells can protect susceptible bacteria from antibiotics in this context. Although the model plasmid in this study declined quickly in populations without selection, the fitness costs of plasmid carriage are within the range seen in other studies^25^ and were more modest in biofilms relative to broth culture.

Detailed modelling of the dynamics of β-lactam resistant and susceptible cells in liquid culture has shown that equilibrium frequencies depend only on dose and cell density.^11^ However, these simple experimental systems do not capture some important effects of bacterial physiology.^12^ β-lactams have bactericidal action for actively dividing cells,^35–37^ so the social protection of susceptible cells in liquid culture must depend on the rapid detoxification of antibiotics by extracellular enzymes.^9–12^ Indeed, extracellular enzyme activity was higher in liquid cultures in our experiments. Unless detoxification is effectively instantaneous, susceptible bacteria must be able to survive an initial pulse of antibiotic exposure while resistant cells detoxify the environment. Alternatively, under equilibrium or chemostat conditions, susceptible cells must be able to tolerate the lower doses of antibiotic produced by the balance between detoxification and diffusion.^38^

Previously, we have argued that these physiological requirements may make the conditions for social detoxification difficult to achieve outside the laboratory. Effective social detoxification in planktonic culture requires that initial doses of antibiotics are not lethal, and that rates of degradation and antibiotic influx do not allow doses to increase until resistant cells are established at high frequencies and densities.^9,12,38^ Our data here, however, show that low frequencies (10–20%) of β-lactamase producers can be very stable in biofilms over a large range of doses. Detoxifying β-lactamases are widespread in the environment^39,40^ and in the clinic,^26,41,42^ so that naturally occurring frequencies could readily provide cooperative resistance in a biofilm if mixed communities were exposed to a pulse of antibiotics.

We predicted that the higher tolerance of antibiotics in biofilms would be key to facilitating indirect fitness benefits from detoxification of β-lactams. There was substantial support for this interpretation of our data in this study, although the hypothesis was hard to test directly. As has been found previously, very high doses of antibiotics (>1 mg/ml) and long periods of exposure (>48 h) were required to kill genotypically susceptible cells in biofilms,^16^ while cells recovered from biofilms were more tolerant of antibiotic exposure than cells recovered from broth culture. This meant that susceptible cells could survive for relatively long periods (>24 h) while resistant cells detoxified the environment. In contrast, much lower doses are lethal over this time period in broth (100 μg/ml), and minimal inhibitory concentrations of ampicillin for laboratory strains of *E. coli* are typically <10 μg/ml.^9–12^ Additional work has confirmed that the proportion of fully dormant persisters in biofilms in this system is <1%.^33^ This indicates that full dormancy plays only a minor role in tolerance of antibiotics in this systems, as might be expected from the existence of multiple tolerance mechanisms in *E. coli*.^21,43^

Biofilms are complex and increased tolerance of antibioitcs covaries with a number of factors (density, presence of extracellular matrix, physiology) and so we cannot be certain that increased protection of sensitive cells in biofilms is due to increased tolerance. Alternative explanations for the enhanced survival of susceptible cells in biofilms include a slow rate of diffusion of β-lactams into biofilms, or a high abundance of extra-cellular β-lactamases.^30^ β-lactams are small molecules, however, and can readily penetrate biofilms,^34^ as we have shown in this system with fluorescently tagged drugs and microscopy.^33^ Moreover, while we found substantial extra-cellular β-lactamase levels in liquid media in this study, a much greater proportion was retained intra-cellularly in biofilms. Potentially, the concentrated spatial distribution of β-lactamases in biofilms (rather than altered total concentration) might provide additional protection, or indeed the assays reported here might underestimate the proportion of extracellular β-lactamases if these enzymes are trapped in the biofilm matrix. Nevertheless, this and other studies have shown that broth cultures of *E. coli* have very high levels of extra-cellular β-lactamases.^11,13^ In addition, as it took over 48 h for susceptible cells to be killed in biofilms, even at 1000 µg/ml of ampicillin, there was ample opportunity for β-lactamases to detoxify the biofilm before the susceptible cells died. Since this increased tolerance is sufficient to explain the observed increased protection, and there is no evidence to support alternative hypotheses, it remains the most likely explanation.

In general, social evolution theory predicts that public goods traits will show negative frequency dependence when there is significant spatial structure separating cheaters and cooperators.^5,44,45^ We, however, observed negative frequency dependence in broth as well as in mixed biofilms, presumably because detoxification resistance yields fitness benefits that are both private (intracellular detoxification) and public (environmental detoxification). In other words, detoxification appears to be a snowdrift game, in which resistant cells (cooperators) outcompete susceptible cells (cheats) even when the frequency of cooperators is low.^46,47^ Similar negative frequency dynamics are seen in detoxifying mercury resistance.^48^

The clinical and environmental implications of this negative frequency dependence could be profound in terms of generating stability in resistance frequencies. Most importantly, these data imply that using β-lactam antibiotics to treat biofilm-focused Gram-negative infections might select for resistance only up to a certain threshold frequency. The prediction of easily attained low-equilibrium frequencies of β-lactam resistance depends on there being a reasonably high cost of resistance, as well as significant within-host or local competition (i.e. low relatedness) between resistant and susceptible cells, an assumption that is hard to validate with current data.^49^ Within-host competition is complicated by the fact that resistance genes, and the plasmids that carry them, are not randomly distributed across bacterial genotypes. For example, in *E. coli*, multi-drug-resistant strains and multi-resistant plasmids are mostly located in a relatively narrow set of nosocomial specialist lineages such as ST131.^50^ Thus mixed infections of commensal *E. coli*, or even varied plasmid carriage within genotypes within hosts,^51^ may not translate to diversity at the site of infection because selection on pathogenic traits and bottlenecks within hosts reduce diversity over the course of infection.^52,53^

The implications of this work for evolution of resistance in a clinical context are still speculative. However, social interactions are predicted to produce a low-equilibrium frequency of resistance driven by frequency-dependent selection, while weaker social interactions such as those imposed by the use of β-lactamase inhibitors, are expected to lead to more effective selection for resistance.^11^ There is increasing evidence for the existence of negative frequency-dependent selection in *E. coli*, particularly for genes encoded on mobile elements,^54^ although definitive data on social protection from β-lactam antibiotics in vivo are still lacking.

## Methods

### Growth conditions

Colony biofilms of a recently isolated faecal *E. coli* (see below) were grown on semipermeable, polycarbonate membranes (Whatman Track-Etched membranes, 25 mm diameter, 0.2 μm pore size). These biofilms were firmly attached to the membranes and could be readily transferred between agar plates.^55^ Starting cultures for transfer experiments were prepared in 5 ml Terrific Broth (Sigma Aldrich) containing 8 µg/ml cefotaxime for the resistant strain and 20 μg/ml chloramphenicol for the engineered susceptible strain. All other cultures used Lysogeny Broth (LB). Ampicillin (Sigma Aldrich) was used as a β-lactam antibiotic at varying doses for transfer experiments. Phosphate buffered saline (PBS) (Sigma Aldrich) was used for biofilm disruptions and serial dilutions. 1 mM isopropyl β-d-1-thiogalactopyranoside (IPTG) was added to the LB agar plates (LBA) and LB, in order to induce the expression of fluorescence. Inocula were prepared by diluting the starting cultures to density of 10^7^ CFU/ml and mixing as appropriate to achieve target initial proportions. Biofilms were grown by inoculating filter membranes on LBA plates (2% w/v) with 5 µl of starting cultures. All cultures were incubated at 37 °C, and all liquid cultures were agitated at 120 rpm.

### Characterization and isolation of WT biofilm forming *E. coli*

In order to identify a strongly biofilm-forming WT strain, 50 isolates of *E. coli*^51^ were screened for biofilm formation in liquid media on micro-titre plates with a commonly used crystal violet assay.^56^ These isolates were recovered from the faeces of healthy outdoor grazing cattle.^51^ From this preliminary screen, 13 isolates were examined in more detail in three replicate experiments (three wells per isolate per experiment) and compared to a laboratory strain (DH10β) and the probiotic *E. coli* Nissle 1917, which were expected to be weak and strong biofilm formers, respectively.

Isolate cc11-1, identified as a strong biofilm former (Fig. 1), was transformed with a plasmid (pE2-Crimson, Clontech) that carried a red fluorescent protein; this plasmid was engineered to express the *bla*_*CTX-M-14*_ gene coding for an extended spectrum β-lactamase (ESBL). Images taken with CLSM used the cc11-1CrimCTXM strain and a plasmid-free competitor cc11-1CyanΔLacZ. The latter strain carried a green fluorescent protein gene from the Clontech plasmid pAmCyan inserted into the *Lac Z* gene (Supplementary Methods). Details of fluorophores are provided in Supplementary Table 1. Cloning and genetic transformation used the Xercise protocol,^57^ details of primers and reaction conditions are provided in the Supplementary Methods and Supplementary Tables 2–7. The WT and the cc11-1CrimCTXM strain were used in all transfer experiments. Plasmid carriage of pCRIMCTXM imposed fitness costs in both marked and unmarked backgrounds (mean fitness cc11-1CrimCTXM versus WT = 0.63, SE 0.01; fitness cc11-1CrimCTXM versus cc11-1CyanΔLacZ = 0.86, SE = 0.02).

### Antibiotic tolerance

Fresh, 24 h biofilms were disrupted by vortexing in 10 ml PBS (Sigma P4417-100AT). A subsample of each disrupted biofilm was diluted in PBS and plated onto LBA to assess baseline cell density; a second sub-sample of each biofilm was exposed to antibiotic for 2 h at 37 °C before being dilution-plated onto LBA. This experiment was repeated twice with either 1000 μg/ml or 100 μg/ml ampicillin (Sigma cat. no. A0166) as the antibiotic challenge (*n* = 8 per dose). This tolerance assay was repeated for broth culture, grown in LB for 24 h (5 ml cultures, 37 °C with shaking at 180 rpm), except that cells were pelleted by centrifugation at 3000 × *g* for 10 min before being re-suspended in PBS (*n* = 4 per dose). The difference in cell counts before and after exposure to ampicillin was used to estimate the proportion of cells tolerant of antibiotics in these conditions.

### Transfer experiments

In order to examine the social interactions between β-lactam-resistant and susceptible strains, we tested whether ampicillin-susceptible cells (WT cc-11-1) could persist alongside detoxifying, resistant cells (cc-11-1CrimCTXM) over a range of doses of ampicillin. The fluorescence plasmid that was engineered to express resistance readily declined in populations through a combination of segregation and fitness costs in the absence of selection pressure. Since we were interested in the dynamics of costly plasmid-associated resistance we designed serial transfer experiments to determine the equilibrium frequency of resistant bacteria in both broth and biofilms under a range of conditions, and characterized competitive dynamics during transfers. We imposed four microbial treatments: pure resistant (R) or susceptible cultures (S), and mixtures with either a low (LR) or high (HR) frequency of resistant cells. These had mean proportion of 0.49 ± 0.02 (SE) in HR and 0.02 ± 0.01 in LR. The same inocula were used for both broth and biofilm experiments. Note that the first transfer is in effect a simple competition experiment with two frequencies and single genotype controls. Experiments ran for five transfers. Cell densities and proportions of each strain were assessed at set-up and before each transfer by serial dilution and plating on LB agar and LB with 8 µg/ml cefotaxime.

Broth transfer experiments used 10-fold dilutions (into 900 µl of LB) every 24 h in 24-well plates with ampicillin at a concentration of 0, 100 or 1000 µg/ml. Biofilms were grown for 24 h before they were transferred to new plates with ampicillin at 0, 500, 1000, 2000 µg/ml for another 24 h. The lower dose of 100 µg/ml was not used on biofilms, since this had limited impact on cell abundance of susceptible bacteria. At this stage the biofilms were disrupted by vortexing in 10 ml PBS. Disrupted biofilms were used to assess cell density and the proportion of resistant cells, and to inoculate new membranes. The transfer experiments were repeated twice both for liquid and biofilm cultures, with three replicates per treatment combination (dose/initial frequency of resistance) in each experiment (*n* = 6 overall).

Exposure to antibiotics for 24 h was strongly inhibitory for susceptible biofilm cultures, but not entirely lethal. Since social protection can be limited to sub-lethal antibiotic exposure^30^ we therefore imposed longer, 3-day antibiοtic exposures to provide fully lethal doses to test whether the switch to lethal conditions affected cheat/cooperator dynamics. These lethal dose experiments used only two doses of ampicillin (0 or 1000 µg/ml) and the four microbial treatments above (S, R, HR or LR) with three replicates. Biofilms on membranes were transferred onto new plates daily to maintain high antibiotic titres and avoid nutrient limitation.

### Microscopy

In order to explore the fine scale spatial structure of plasmid carrying and plasmid-free bacteria, we performed competitions on filter discs with plasmid carriers cc11-1CrimCTXM and chromosomally marked strains cc11-1CyanΔLacZ. We inoculated filter discs at two ratios: 50:50 and 90:10 mixtures of plasmid carriers:plasmid-free cells, respectively. We imaged these biofilms under two experimental conditions: first 24 h-old biofilms formed directly from fresh broth and second, biofilms re-formed after a single transfer from disrupted biofilm. These treatments reflect conditions at the set-up, and during, the transfer experiments. Biofilms were mounted on glass slides in immersion oil to prevent desiccation. Biofilms were imaged using a confocal fluorescence microscope (Leica AF6000) with the following settings: objective ×20 plan apochromatic; cyan excitation wavelength 448 nm–cyan emission wavelength 483/510 nm; red excitation wavelength 552 nm–red fluorescence detector 633/660 nm. Image processing and analysis used Leica software (LAS X version 3.5.1.18803) to generate orthogonal sections over whole biofilms of approximate dimensions: *x*,*y* = 581.25 µm, *z* ≈ 250 µm. Imaging was carried out for at least three biofilms in each treatment, detailed microscope settings are provided in the Supplementary Methods. Experiments were repeated with bacteria marked with similar plasmid, and showing similar growth rates, these experiments also examined fine scale structure and are described in the Supplementary Methods.

### β-lactamase activity in liquid and biofilm cultures

In *E. coli* β-lactamases are typically exported to the periplasmic space^58^ but can also undergo extracellular export via outer membrane vesicles. In either location they are available to detoxify media and provide shared benefits to genetically susceptible cells.^59^ We used nitrocefin-based colorimetric assays to test whether the variation in the fraction of extracellular activity could explain the different levels of social protection across broth and biofilms. Broth cultures (grown for 24 h) were centrifuged at 3000×*g* for 15 min. The supernatant was used for measuring the activity of the extracellular β-lactamases and the pellet was stored at −20 °C awaiting processing. To measure the intracellular activity, the pellet was re-suspended in 500 µl PBS and incubated at 37 °C with 1 mg/ml lysozyme and 2 mM EDTA for 1 h. For the measurements, 2 µl of the sample was mixed with 198 µl PBS and 20 µg nitrocefin in 96-well plates and placed in a spectrophotometer for 10 min before recording the OD_486_. For the low resistant and susceptible cultures grown on LB, 20 µl samples was mixed with 180 µl PBS to compensate for the low enzyme activity. Treatments were replicated three times. The results were corrected for sample dilution before analysis. Biofilms were grown under our standard conditions (see above) and after a 24 h exposure to 1000 µg/ml ampicillin. Biofilms were disrupted in 5 ml PBS and centrifuged at 3000 × *g* for 15 min and the supernatants were used to measure extracellular activity, as above. Intracellular activity was recorded as above except that the pellet was re-suspended in 5 ml PBS and 20 µl samples were diluted with 180 µl PBS. For both broth and biofilms, subsamples of pellets were dilution-plated on LB agar prior to freezing in order to assess enzymatic activity per colony forming unit.

### Data analysis

Optical density data from crystal violet assays were analysed as mixed effect models with replicates nested within experiment, after square root transformation. The proportion of resistant bacteria in the population was calculated by scoring fluorescent and non-fluorescent colonies on LB and by plating on selective media (LB with 8 µg/ml cefotaxime). The estimated proportion of resistant colonies resulting from counts on LB plates and antibiotic-containing plates were very similar (Supplementary Fig. 4) but were averaged for robustness. Relative fitness was calculated as the ratio of Malthusian parameters,^60^ and the results of the first transfer therefore represent a simple competition experiment. In order to make comparisons between treatments with one and two genotypes (or when densities were undetectable) we also calculated the reproductive rate in bacterial doublings in the first transfer, instead of relative fitness. We transformed final counts by adding the minimum detectable density in order to deal with zero counts so that reproductive rate = [log (no. of susceptible cells at the final point + 2.5 × 10^6^) − log(no. cells at the initial point)]/log_2_.

Statistical analysis was performed with Rv3.4.3.^61^ Package nlme^62^ was used for the linear mixed effects models. Fixed and random effect residuals were checked for normality and homoscedasticity by graphical methods and data transformations (square root for bacterial abundance, arc-sine for proportions, log_10_ for relative fitness) were chosen accordingly. After the significance of fixed variables was checked, non-significant terms were removed by model simplification and likelihood ratio tests, or on the basis of the Akaike Information Criterion (AIC) if removal did not alter residual degrees of freedom.

### Reporting summary

Further information on research design is available in the Nature Research Reporting Summary linked to this article.

## Supplementary information

Supplementary Materials

Reporting Summary Checklist

## Data Availability

The raw data that support this study are available at the University of Exeter institutional repository ORE with the identifier 10.24378/exe.1943. Figures 1, 3–6 have available raw data. Unprocessed images in Fig. 2 are available on request. Biological materials are available on request from the corresponding author.
