# Supplementary material for: Biofilms facilitate cheating and social exploitation of β-lactam resistance in Escherichia coli
Source: NPJ Biofilms Microbiomes. 2019 Nov 29;5:36. doi: 10.1038/s41522-019-0109-2 (PMC6884583; doi:10.1038/s41522-019-0109-2)

Supplementary Information file for Amanatidou et al "Biofilms facilitate cheating and social exploitation of β-lactam resistance in *Escherichia coli*"

Supplementary Methods

Microscope settings for Figure 2

Fig 2a-

<Size>3.91 MB
<Channels>
ChannelTag="3" Resolution="8" NameOfMeasuredQuantity="" Min="0.000000e+000" Max="2.550000e+002" Unit="" LUTName="" IsLUTInverted="0" BytesInc="0" BitInc="0">
ChannelTag="2" Resolution="8" NameOfMeasuredQuantity="" Min="0.000000e+000" Max="2.550000e+002" Unit="" LUTName="" IsLUTInverted="0" BytesInc="1" BitInc="0">
ChannelTag="1" Resolution="8" NameOfMeasuredQuantity="" Min="0.000000e+000" Max="2.550000e+002" Unit="" LUTName="" IsLUTInverted="0" BytesInc="2" BitInc="0">
<Dimensions>
<DimensionDescription DimID="X" NumberOfElements="1141" Origin="0" Length="1,141" Unit="m" BitInc="0" BytesInc="3" UnitOrigin="m" EndPosition="1,141" UnitEndPosition="m" LogicalUnit="pixels" Voxel="1.001">
<DimensionDescription DimID="Y" NumberOfElements="1141" Origin="0" Length="1,141" Unit="m" BitInc="0" BytesInc="3423" UnitOrigin="m" EndPosition="1,141" UnitEndPosition="m" LogicalUnit="pixels" Voxel="1.001">

Fig 2c

<Size>6.01 MB</Size>
<Channels>
ChannelTag="3" Resolution="8" NameOfMeasuredQuantity="" Min="0.000000e+000" Max="2.550000e+002" Unit="" LUTName="" IsLUTInverted="0" BytesInc="0" BitInc="0">
ChannelTag="2" Resolution="8" NameOfMeasuredQuantity="" Min="0.000000e+000" Max="2.550000e+002" Unit="" LUTName=""

IsLUTInverted="0" BytesInc="1" BitInc="0">
ChannelTag="1" Resolution="8" NameOfMeasuredQuantity=""
Min="0.000000e+000" Max="2.550000e+002" Unit="" LUTName=""
IsLUTInverted="0" BytesInc="2" BitInc="0">
<Dimensions>
<DimensionDescription DimID="X" NumberOfElements="1415" Origin="0"
Length="1,415" Unit="m" BitInc="0" BytesInc="3" UnitOrigin="m"
EndPosition="1,415" UnitEndPosition="m" LogicalUnit="pixels" Voxel="1.001">
<DimensionDescription DimID="Y" NumberOfElements="1415" Origin="0"
Length="1,415" Unit="m" BitInc="0" BytesInc="4245" UnitOrigin="m"
EndPosition="1,415" UnitEndPosition="m" LogicalUnit="pixels" Voxel="1.001">

Fig2 panels b,e

<Size>5.93 MB</Size>
ChannelDescription
ChannelTag="3" Resolution="8" NameOfMeasuredQuantity=""
Min="0.000000e+000" Max="2.550000e+002" Unit="" LUTName=""
IsLUTInverted="0" BytesInc="0" BitInc="0"
ChannelTag="2" Resolution="8" NameOfMeasuredQuantity=""
Min="0.000000e+000" Max="2.550000e+002" Unit="" LUTName=""
IsLUTInverted="0" BytesInc="1" BitInc="0">
ChannelTag="1" Resolution="8" NameOfMeasuredQuantity=""
Min="0.000000e+000" Max="2.550000e+002" Unit="" LUTName=""
IsLUTInverted="0" BytesInc="2" BitInc="0">
<Dimensions>
<DimensionDescription DimID="X" NumberOfElements="1406" Origin="0"
Length="1,406" Unit="m" BitInc="0" BytesInc="3" UnitOrigin="m"
EndPosition="1,406" UnitEndPosition="m" LogicalUnit="pixels" Voxel="1.001">
<DimensionDescription DimID="Y" NumberOfElements="1406" Origin="0"
Length="1,406" Unit="m" BitInc="0" BytesInc="4218" UnitOrigin="m"
EndPosition="1,406" UnitEndPosition="m" LogicalUnit="pixels" Voxel="1.001">

Fig 2 d,f

<Size>5.62 MB
<Channels>
ChannelTag="3" Resolution="8" NameOfMeasuredQuantity=""

Min="0.000000e+000" Max="2.550000e+002" Unit="" LUTName=""
IsLUTInverted="0" BytesInc="0" BitInc="0">
ChannelTag="2" Resolution="8" NameOfMeasuredQuantity=""
Min="0.000000e+000" Max="2.550000e+002" Unit="" LUTName=""
IsLUTInverted="0" BytesInc="1" BitInc="0">
ChannelTag="1" Resolution="8" NameOfMeasuredQuantity=""
Min="0.000000e+000" Max="2.550000e+002" Unit="" LUTName=""
IsLUTInverted="0" BytesInc="2" BitInc="0">
<Dimensions>
<DimensionDescription DimID="X" NumberOfElements="1369" Origin="0"
Length="1,369" Unit="m" BitInc="0" BytesInc="3" UnitOrigin="m"
EndPosition="1,369" UnitEndPosition="m" LogicalUnit="pixels" Voxel="1.001">
<DimensionDescription DimID="Y" NumberOfElements="1369" Origin="0"
Length="1,369" Unit="m" BitInc="0" BytesInc="4107" UnitOrigin="m"
EndPosition="1,369" UnitEndPosition="m" LogicalUnit="pixels" Voxel="1.001">

Microscopy confirmation of void existence

Biofilms were stained with 10 μl of a 1000x dilution of stock of Rhodamine 6G chloride (Molecular Probes, Invitrogen), added underneath the polycarbonate membrane for a 2h exposure. This method was chosen in order to minimise the disturbance of the structure that a flow from the top could cause. Excitation for rhodamine was measured at 500 nm (set to 80% intensity) and emission 515-590 nm. In contrast to images in the main MS these experiments used biofilms were grown on black polycarbonate membranes (Sterlitech, PCTB 0225100) for 24 h and then transferred to ampicillin-containing plates (0 or 1000 μg/ml) for another 24 h as before. Five locations in each of five biofilms were imaged for each type of biofilm. Imaging was performed with a Leica SP5X upright microscope, equipped with a super continuum laser source set to 75% intensity. The system was controlled by the software LAS AF ver. 2.6.1. Images were recorded with a long working distance air lens 63x NA 0.7 in order to avoid any disturbance of the biofilm. Excitation at 611 nm (80% intensity) was used for the crimson protein and 475 nm (100% intensity) for the cyan protein. Emission for the crimson protein was measured between 625 – 725 nm and for the cyan protein between 490 – 550 nm. Imaris 8.1.2 (Imaris, Bitplane AG, available at http://bitplane.com) and JImageAnalyser 1.4, an in-house version of

ImageJ at UFZ Magdeburg, were used for thresholding (Table S1), and Imaris was also used for image rendering. Images are shown in Supplementary Figure S1.

Bacterial strains and plasmids

E. coli DH10β (for controls and propagation) and cc11-1 (environmental isolate) were transformed with the constructed plasmids described below. The pCT plasmid carrying the $bla_{CTX\text{-}M\text{-}14}$ gene was provided by the Animal Health and Veterinary Laboratories Agency (AHVLA, Weybridge, UK) [Cottell et al 2011, Emerg Infect Dis v17, p645]. Plasmid pTopo carrying the dif-CAT (chloramphenicol gene) cassette was provided by Cobra Biomanufacturing [Bloor & Cranenburgh, 2006; AEM v72, p2520]. The fluorescent vectors pAmCyan and pE2-Crimson, were bought from Clontech (Cat no 632440 and 632553 respectively). The properties of the fluorescent proteins carried by these vectors can be found in Table S1.

Plasmid construction and integration of markers into chromosome. The antibiotic resistance genes ($bla_{CTX\text{-}M\text{-}14}$ and CAT) and the modified vectors were amplified with their respective native promoters by PCR. *Sac*I restriction sites were added at both ends. Digestion and ligation reactions are described below. The Clontech fluorescent vectors were used as the backbone vector with all their original elements intact (origin of replication, lac promoter for the fluorescence gene and the fluorescence gene), except for part of the ampicillin resistance gene (Amp^r), which was removed and replaced by $bla_{CTX\text{-}M\text{-}14}$ or CAT gene. The modified backbone vectors were obtained with PCR with *Sac*I restriction sites added at both ends. For further details on PCR primers, cycles, reaction components see Supplementary Tables 2 – 6. All PCR products were confirmed with gel electrophoresis.

Mutants with a chromosomal integration of a fluorescence marker (cc11-1CyanΔLacZ) were constructed in order to facilitate imaging of the spatial structure of biofilms containing cells with and without plasmids. Primers bearing homology to the *LacZ* gene were used to amplify the region of the pCyanCTXM plasmid encoding $bla_{CTX\text{-}M\text{-}14}$ and the cyan fluorescence gene (Table S3). As above gene replacement used the Xer-

cise protocol [Bloor & Cranenburgh, 2006; AEM v72, p2520] using Q5-polymerase. PCR constructs were gel purified for insertion by electroporation into competent cc-11-1 cells, previously transformed with the helper plasmid pLGBE. Transformants were grown on LB plates containing IPTG and cefotaxime (8μg /ml) at 37°C. Confirmation of transformants used colony PCR with NEB Taq (Cat No M0273) (primers in Supplementary Table 7). Stability of the insertion was tested via overnight culture in LB and the relative fitness of chromosomal integrants and cells carrying pCRIMCTXM was calculated in overnight cultures in LB broth using the methods described for the liquid culture in the main MS.

Digestions and ligations. All PCR products (inserts and vectors) were cleaned up with the Promega Wizard SV Gel and PCR Clean-Up System (Cat no A9281) before digesting with enzyme *Sac*I-HF, chosen because this restriction site was not present in any of the vectors or inserts. Typical digestion volumes were 50 μl (to avoid star activity), containing 0.5 -1 μg DNA. The reactions were incubated at 37 °C for 30 min to 1.5 h. The vector was dephosphorylated with an alkaline phosphatase that was added into the vector digestion reaction after the first 30 min and the whole reaction was incubated at 37 °C for another 15 min. The digestions were cleaned up before proceeding to ligations with the same Promega cleanup system mentioned above. Typical vector: insert ratios were 1:2, 1:3, 1:5, or 1:6. The ligations were incubated at room temperature for a time period of 2 h to 4 h and then inactivated as per the enzyme manufacturer's instructions. All enzymes used can be found in Table S3. PCR primers and cycle conditions can be found in Tables S4-S6.

Plasmid purifications and bacterial transformations.
Plasmid pCT was isolated using the Qiagen Highspeed midi plasmid kit (Qiagen, UK, Cat no 12643). Plasmid pTopo and the subsequently engineered plasmids (crimson or cyan with *bla*CTX-M-14 or CAT) were isolated with the Qiagen Highspeed mini plasmid kit (Qiagen UK, Cat no 27104). The engineered plasmids were transformed into DH10β-cells with electroporation using the high efficiency electro-transformation protocol for *Escherichia coli*, suggested by Bio-Rad

(http://www.bio-rad.com/webroot/web/pdf/lsr/literature/4006174B.pdf). The transformed cc11-1 strains were recovered on IPTG-containing LB plates with the appropriate antibiotic selector. IPTG was used for inducing the transcription of the fluorescence gene. PCR was used for the confirmation of the transformants.

Sequencing constructed plasmids

The insertion in the plasmids was confirmed by sequencing. The constructed, cloned plasmids were purified from *E.coli* DH10*β* with the Qiagen Highspeed mini plasmid kit (Qiagen UK, Cat no 27104) and sent to Eurofins Genomics for sequencing. The sequencing primers used can be found in Table S7.

Supplementary Tables

Supplementary Table 1 Properties of the fluorescent proteins used in experiments

Vector	pE2-Crimson	pAmCyan
Origin of replication	pUC	pUC
Selective gene	Ampicillin	Ampicillin
Fluorescent protein Promoter	lacZ	lacZ
Fluorescent Protein	E2-Crimson	AmCyan 1
Protein Origin	DsRed-Express2	*Anemonia majano*
Excitation max	611 nm	458 nm
Emission max	646 nm	489 nm

Supplementary Table 2: NEB Enzymes used for PCRs, digestion, dephosphorylation and ligation reactions

Enzyme	Function
Q5 high fidelity polymerase (M0491S)	PCR (genes & vector)
OneTaq hot start high fidelity polymerase (M0493S)	Confirmation colony PCR
SacI-HF restriction enzyme (R3156S)	Digestions
T4 DNA ligase (M0202S)	Ligations

Antarctic phosphatase (M0289S)	Plasmid dephosphorylation

Supplementary Table 3: All primers used for cloning. Homology in primer sequences are shown in red.

Primers	Sequence	Function	Product size	Template
CATSacIF1 (1226)	5'AAAAGAGCTC GGCGTAGCACCA GGCGTT	CAT gene – forward primer	1026bp	pTOPOdif
CATSacIR1 (2251)	5'AAAAGAGCTC GGCCGCCAGTGT GATGGA	CAT gene – reverse primer		
CTXSacIF1 (69329)	5'AAAAGAGCTC ACGGTCTGCGTT GTCGGG	CTX-M gene– forward primer	1463bp	pCT
CTXSacIR1 (71242)	5'AAAAGAGCTCC TGCAAACGGTGC TGCGG	CTX-M gene– reverse primer		
CyanSacF4 (2177)	5'AAAAGAGCTCT TCTGCGCTCGGC CCTTC	Cyan/ Crimson backbone – amp out forward primer	2930bp	pAmCyan/ pE2-Crimson
CyanSacR4 (1781)	5'AAAAGAGCTCT GCCCGGCGTCAA TACGG	Cyan/Crimson backbone– amp out reverse primer		
Lac_CyanCT XM F	5' - ATTGCGGCCTAT ATGGATGTT GGAACCGTAAGA GAACCCGA CTGGAAAGCGGG CAG	forward for insertion of CyanCtxm in the lacZ promoter	3707bp	pCyanCT XM
Lac_CyanCT XM R	5' - GCGGGCAGTGAG CGCAACGC AATTAATGTGAG TTAG CTGCAAACGGTG CTGCGG	reverse for insertion of CyanCtxm in the lacZ promoter		

Supplementary Table 4: PCR conditions for obtaining the *bla* $_{CTX-M-14}$ gene and the modified backbone vectors

Step	Temperature	Time
Initial denaturation	98	1min
Denaturation	98	10sec
Annealing	70	30sec
Extension	72	40 sec
Repeat steps 2-4 for 34 times		
Final extension	72	2min
Incubation	10	Forever

Supplementary Table 5: PCR conditions for obtaining the *CAT* gene

Step	Temperature	Time
Initial denaturation	98	1min
Denaturation	98	10sec
Annealing	71	30sec
Extension	72	40 sec
Repeat steps 2-4 for 34 times		
Final extension	72	2min
Incubation	10	Forever

Supplementary Table 6: Typical PCR reaction for a final volume of 25µl per reaction (double quantities were used for a final reaction volume of 50µl)

Components	Quantities (µl)	
DNA	1.0	1.0
dNTPs [10mM]	0.5	0.5
Forward primer [10µM)	1.25	1.25
Reverse primer [10µM)	1.25	1.25
DNA Polymerase (Q5)	0.25	0.25
Polymerase buffer	5.0	5.0
Polymerase enhancer	-	5.0
Water	15.75	10.75

Supplementary Table 7: Primers used for confirming the insertion of the antibiotic resistance genes in the constructed plasmids or chromosome of cc11-1 by sequencing

Primer	Sequence	Plasmid/target
pCrimCTXM-3819R	5'-GAT ACG GGA GGG CTT ACC A	CrimCTXM
pCrimCTXM-1319F	5'-GGC CTC GTG ATA CGC CTA TT	CrimCTXM
pCyanCAT-2833R	5'-GAT ACG GGA GGG CTT ACC A	CyanCAT
pCyanCAT-1333F	5'-GGC CTC GTG ATA CGC CTA TT	CyanCAT
F Lac diag	5'-ACG GAA AGA GTA ACG TTG GGT GC	chromosomal insertion of CyanCTX_M
R Lac diag	5'-GCG CCA TTA CCG AGT CCG GG	chromosomal insertion of CyanCTX_M

Supplementary Figure 1: Images from 3-dimensional multichannel confocal laser scanning microscopy data sets of four different colony biofilms of engineered cc11-1 containing different proportions of resistant and susceptible cells. Resistant, High resistant, Low resistant and Susceptible indicate initial frequencies of 100, 50, 2 and 0% cc-11-1 CrimCTXM. The biofilms were grown for 48 h on polycarbonate membranes. b-lactam-resistant CrimCTXM (red) and susceptible CyanCAT (green) cells are shown in the top row. Rhodamine-stained images (grey scale) are shown in the second row, with the flat grey colour indicating voids, and an overlay of all three channels are shown in the third row. The scale bars equal 10 µm. Imaris Isosurfaces were used to enhance image presentation. Grid size = 10 µm.

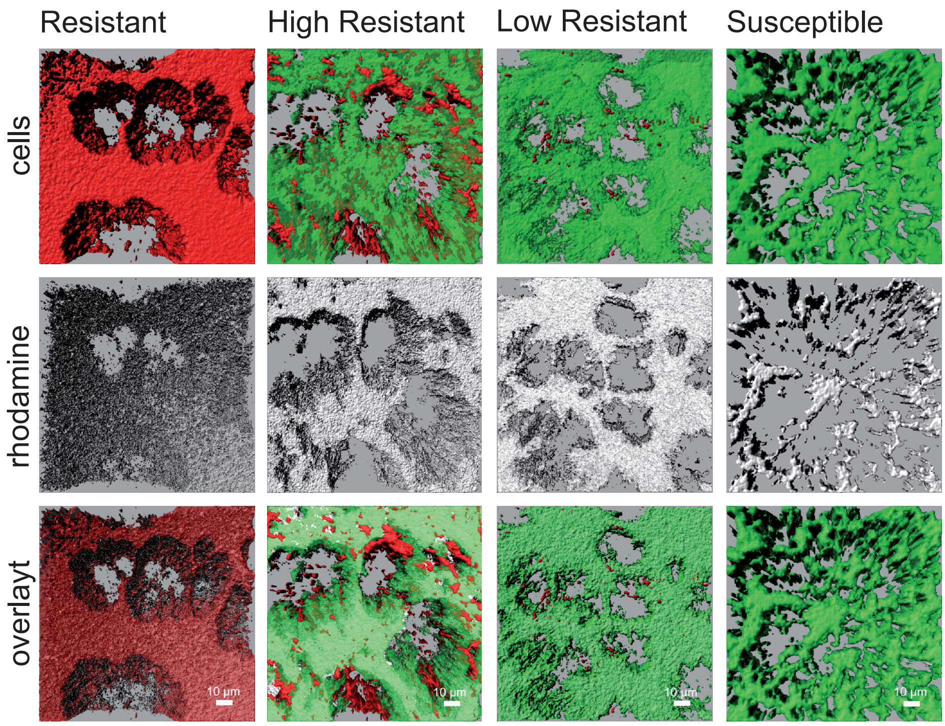

Supplementary Figure 2: Resistant reproductive rate measured as number of doublings over transfer 1, as a function of antibiotic dose and proportion of susceptible cells in mixtures. In all panels x-axis is categorical

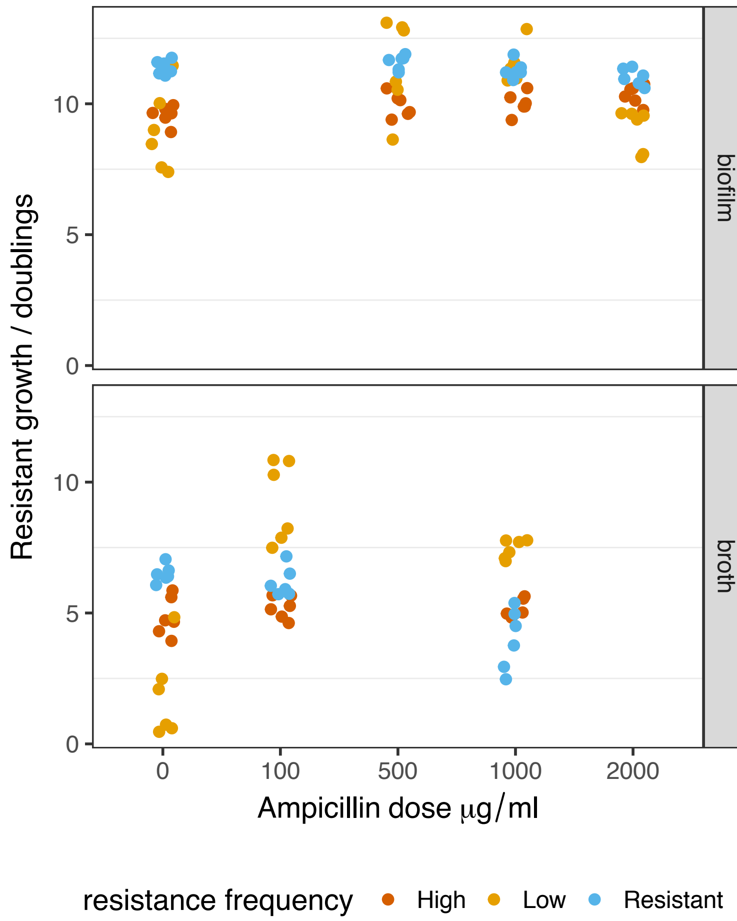

Supplementary Figure 3. **a.** Population dynamics and **b.** relative fitness of susceptible cells in biofilms in the presence and absence of ampicillin- biofilms were grown for three days at 1000 μg / ml in order to impose lethal conditions on susceptible cells. Time point -1 represents the initial inoculum, and time point 0 represents the biofilm established prior to exposure to ampicillin. In biofilms containing only susceptible bacteria, no viable cells remain after three days of exposure to this concentration of antibiotic. The horizontal line at $W = 1$ indicates equal fitness of resistant and susceptible bacteria.

(A)

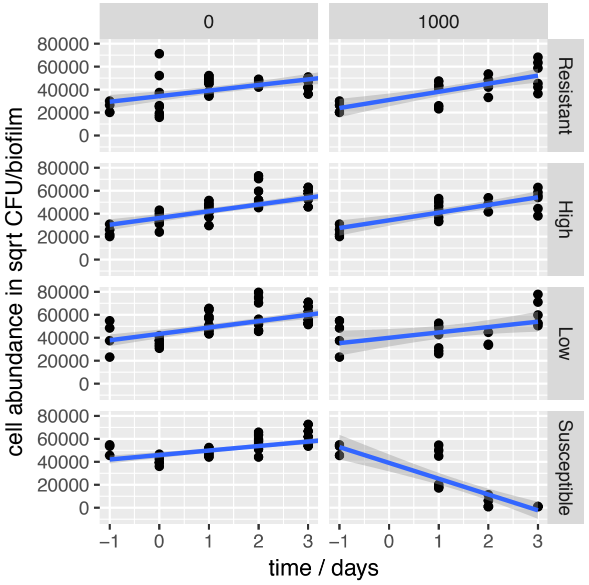

(B)

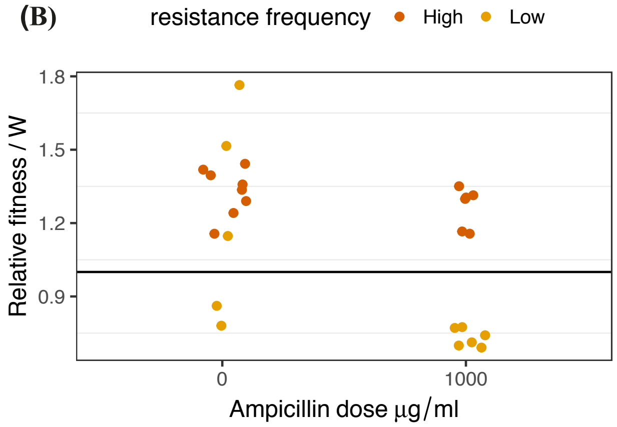

Supplementary Figure 4. Comparison of different methods of scoring proportion of resistant cells recovered from passage experiment: (A) proportion of cefotaxime resistant cells to total bacteria recovered on LB agar without antibiotics (B) and proportion of red fluorescent cells recovered on LB agar plates, indicating carriage of the pE2_CrimCTXM resistant vector.

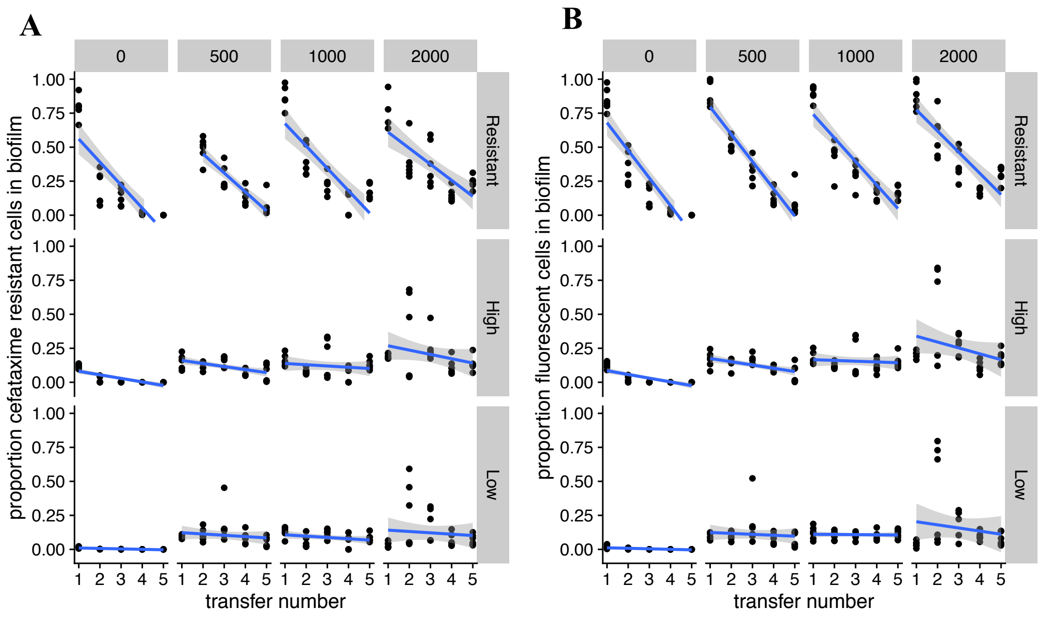

Supplement: Supplementary file 1 — Supplementary Materials [file 41522_2019_109_MOESM1_ESM.pdf]
